# [μ-(3,4,5,6,7-η:1,9,10,11,12)-5,11-Di-*tert*-butyl-2,2,8,8-tetra­methyl-2,8-disila­tricyclo­[7.3.0.0^3,7^]dodeca­tetra­enedi­yl]bis­[dicarbonyl­ruthenium(I)]

**DOI:** 10.1107/S1600536812033454

**Published:** 2012-08-11

**Authors:** Bolin Zhu

**Affiliations:** a College of Chemistry and Tianjin Key Laboratory of Structure and Performance for Functional Molecules, Tianjin Normal University, Tianjin 300387, People’s Republic of China

## Abstract

The title compound, [Ru_2_(C_22_H_34_Si_2_)(CO)_4_], contains two Ru^I^ atoms linked by a bridging (η^5^-^*t*^BuC_5_H_2_)_2_(SiMe_2_)_2_ ligand (^*t*^Bu is a *tert*-butyl and Me is a methyl group) with an Ru—Ru bond length of 2.8401 (7) Å. The dihedral angle between the planes of the cyclo­penta­dienyl rings of the ligand is 123.13 (19)°. The four terminal carbonyl ligands are bound in a symmetrical and staggered array. In the crystal, mol­ecules are linked *via* pairs of C—H⋯O hydrogen bonds, forming inversion dimers.

## Related literature

For structures of non-bridged, singly-bridged, and doubly-bridged bis­(cyclo­penta­dien­yl)ruthenium analogues of the title compound, see: Mills & Nice (1967 ▶); Burger (2001 ▶); Zhou *et al.* (1997 ▶); Bitterwolf *et al.* (1996 ▶); Ovchinnikov *et al.* (2002 ▶); Zhu *et al.* (2012 ▶). For the fulvalene diruthenium carbonyl complex (η^5^:η^5^-C_10_H_8_)Ru_2_(CO)_4_, see: Boese *et al.* (1997 ▶).
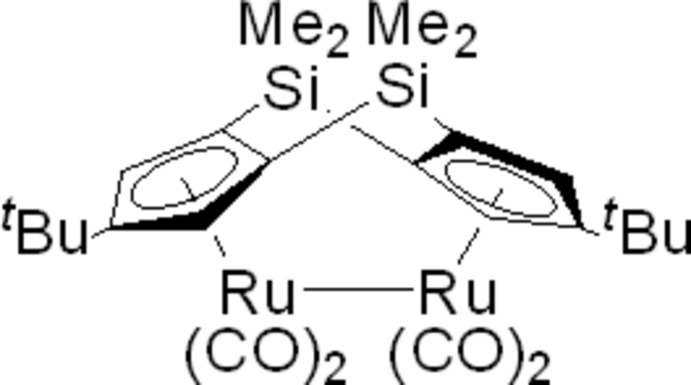


## Experimental

### 

#### Crystal data

[Ru_2_(C_22_H_34_Si_2_)(CO)_4_]
*M* *_r_* = 668.85
Triclinic, 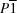

*a* = 10.632 (3) Å
*b* = 10.886 (3) Å
*c* = 14.546 (5) Å
α = 89.518 (5)°
β = 71.581 (4)°
γ = 61.560 (4)°
*V* = 1384.0 (7) Å^3^
*Z* = 2
Mo *K*α radiation
μ = 1.21 mm^−1^
*T* = 173 K
0.17 × 0.16 × 0.15 mm

#### Data collection

Bruker APEXII CCD diffractometer
Absorption correction: multi-scan (*SADABS*; Bruker, 2005 ▶) *T*_min_ = 0.821, *T*_max_ = 0.840
6921 measured reflections
4802 independent reflections
4101 reflections with *I* > 2σ(*I*)
*R*_int_ = 0.029

#### Refinement

*R*[*F* ^2^ > 2σ(*F* ^2^)] = 0.032
*wR*(*F* ^2^) = 0.077
*S* = 1.04
4802 reflections
317 parameters
H-atom parameters constrained
Δρ_max_ = 0.92 e Å^−3^
Δρ_min_ = −0.86 e Å^−3^

### 

Data collection: *APEX2* (Bruker, 2005 ▶); cell refinement: *SAINT* (Bruker, 2005 ▶); data reduction: *SAINT*; program(s) used to solve structure: *SHELXS97* (Sheldrick, 2008 ▶); program(s) used to refine structure: *SHELXL97* (Sheldrick, 2008 ▶); molecular graphics: *SHELXTL* (Sheldrick, 2008 ▶); software used to prepare material for publication: *SHELXTL*.

## Supplementary Material

Crystal structure: contains datablock(s) I, global. DOI: 10.1107/S1600536812033454/su2478sup1.cif

Structure factors: contains datablock(s) I. DOI: 10.1107/S1600536812033454/su2478Isup2.hkl

Additional supplementary materials:  crystallographic information; 3D view; checkCIF report

## Figures and Tables

**Table 1:** Hydrogen-bond geometry (Å, °)

*D*—H⋯*A*	*D*—H	H⋯*A*	*D*⋯*A*	*D*—H⋯*A*
C20—H20*A*⋯O2^i^	0.98	2.60	3.571 (5)	171

Symmetry code: (i) 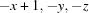.

**Fig. 1.**
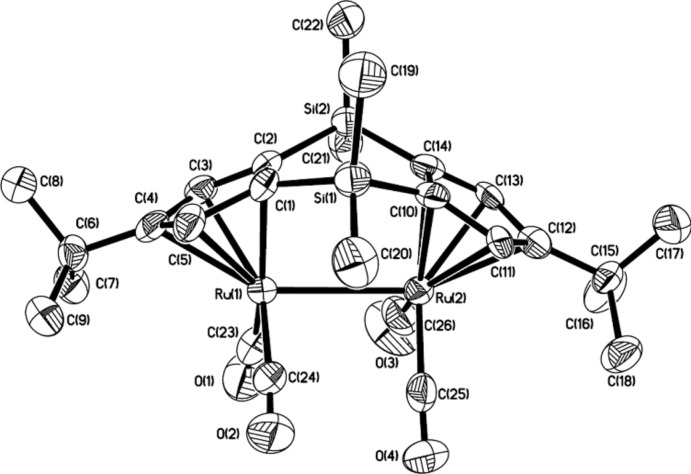
A view of the molecular structure of the title compound with the atom numbering. Displacement ellipsoids are drawn at the 30% probability level.

## supplementary crystallographic information

### Comment

Recently, a series of reactions of doubly-bridged ligand precursors (C_5_H_4_(E))(C_5_H_4_(E')) (E, E' = CH_2_, CMe_2_, SiMe_2_, or GeMe_2_) with Ru_3_(CO)_12_ have been reported by the group of Professor Angelici (Ovchinnikov *et al.*, 2002) and our groups (Zhu *et al.*, 2012), which generally afforded the corresponding doubly-bridged bis(cyclopentadienyl) dinuclear complex containing an elongated Ru—Ru bond. To develop a deeper understanding of the relationship between the structure of the ligand and the Ru—Ru bond distance, and make a comparison of the Ru—Ru bond distance with those in the respective non-bridged and singly-bridged bis(cyclopentadienyl) ruthenium analogues, we carried out the reaction of the doubly-bridged ligand precursor (*^t^*BuC_5_H_3_)_2_(SiMe_2_)_2_ with Ru_3_(CO)_12_ in refluxing xylene, which afforded the expected title product whose crystal structure we report on herein.

The molecular structure of title compound is presented in Fig. 1. It has approximate *C_2v_* symmetry, as reflected in the small torsion angle *D*Cp(centroid)–Ru1–Ru2–Cp(centroid) (ca. 15.8°). The dihedral angle between the planes of the Cp rings of the (η^5^-C_5_H_2_*^t^*Bu)_2_(SiMe_2_)_2_ ligand is rather large, 123.13 (19) °, which results in a longer than normal Ru1–Ru2 single bond distance of 2.8401 (7) Å, longer than that [2.8180 (3) Å] in its parent complex [(η^5^-C_5_H_3_)_2_(SiMe_2_)_2_]Ru_2_(CO)_4_ (Ovchinnikov *et al.*, 2002)_._ Therefore, the two *^t^*Bu substituents on title compound have considerable effect on the geometry of the system.

The elongated Ru—Ru distance makes CO bridging unfavorable. This situation is similar to that in other doubly-bridged analogues, for example 2.8420 (8) Å in [(η^5^-C_5_H_3_)_2_(CMe_2_)(SiMe_2_)]Ru_2_(CO)_4_, 2.824 (1) Å in [(η^5^-C_5_H_3_)_2_(CMe_2_)(GeMe_2_)]Ru_2_(CO)_4_, 2.8382 (9) Å in [(η^5^-C_5_H_3_)_2_(CH_2_)(SiMe_2_)]Ru_2_(CO)_4_, 2.8429 (7) Å in [(η^5^-C_5_H_3_)_2_(CH_2_)(GeMe_2_)]Ru_2_(CO)_4_ (Zhu *et al.*, 2012), and 2.821 (1) Å in the fulvalene diruthenium carbonyl complex (η^5^:η^5^-C_10_H_8_)Ru_2_(CO)_4_ (Boese *et al.*, 1997).

Generally, due to the rigid structure of the doubly-bridged ligand, the Ru—Ru bond distances in the above-mentioned complexes are obviously longer than those in the respective non-bridged and singly-bridged analogues, for example 2.735 (2) Å in *trans*-[(η^5^-C_5_H_5_)Ru(CO)(µ-CO)]_2_ (Mills *et al.*, 1967), 2.7879 (4) Å in (CMe_2_)[(η^5^-C_5_H_4_)Ru(CO)_2_]_2_ (Burger, 2001), or 2.705 Å in (SiMe_2_)[(η^5^-C_5_H_4_)Ru(CO)(µ-CO)]_2_ (Zhou *et al.*, 1997; Bitterwolf *et al.*, 1996).

### Experimental

A solution of (C_5_H_3_*^t^*Bu)(SiMe_2_))_2_ (80 mg, 0.22 mmol) and Ru_3_(CO)_12_ (80 mg, 0.13 mmol) in xylene (20 ml) was refluxed for 15 h. After removal of the solvent under reduced pressure, the residue, which was dissolved in a minimum amount of CH_2_Cl_2,_ the solution was chromatographed on an alumina column using petroleum ether–CH_2_Cl_2_ (5:1) as eluent. A yellow band was eluted and collected. After removal of the solvents under vacuum from the above eluate, the residue was recrystallized from *n*-hexane/CH_2_Cl_2_ (1:1) at 263 K to give colourless crystals of the title compound (54 mg, 36%). Anal. Calcd for C_26_H_34_O_4_Ru_2_Si_2_: C, 46.69; H, 5.12. Found: C, 46.82; H, 5.17. Spectroscopic data for the title compound is given in the archived CIF.

### Refinement

All the hydrogen atoms could be located in difference electron density maps. In th final cycles of refinement they were included in calculated positions and treated as riding atoms: C-H = 0.98 and 1.00 Å for CH_3_ and CH H-atoms, respectively, with U_iso_(H) = k × U_eq_(parent C-atom), where k = 1.5 for CH_3_ H-atoms and = 1.2 for other H-atoms.

### Crystal data

**Table:**

[Ru_2_(C_22_H_34_Si_2_)(CO)_4_]	*Z* = 2
*M**_r_* = 668.85	*F*(000) = 676
Triclinic, *P*1	*D*_x_ = 1.605 Mg m^−^^3^
Hall symbol: -P 1	Mo *K*α radiation, λ = 0.71073 Å
*a* = 10.632 (3) Å	Cell parameters from 3308 reflections
*b* = 10.886 (3) Å	θ = 2.4–28.3°
*c* = 14.546 (5) Å	µ = 1.21 mm^−^^1^
α = 89.518 (5)°	*T* = 173 K
β = 71.581 (4)°	Block, yellow
γ = 61.560 (4)°	0.17 × 0.16 × 0.15 mm
*V* = 1384.0 (7) Å^3^	

### Data collection

**Table:**

Bruker APEXII CCD diffractometer	4802 independent reflections
Radiation source: fine-focus sealed tube	4101 reflections with *I* > 2σ(*I*)
Graphite monochromator	*R*_int_ = 0.029
φ and ω scans	θ_max_ = 25.0°, θ_min_ = 1.5°
Absorption correction: multi-scan (*SADABS*; Bruker, 2005)	*h* = −12→11
*T*_min_ = 0.821, *T*_max_ = 0.840	*k* = −12→6
6921 measured reflections	*l* = −17→17

### Refinement

**Table:**

Refinement on *F*^2^	Primary atom site location: structure-invariant direct methods
Least-squares matrix: full	Secondary atom site location: difference Fourier map
*R*[*F*^2^ > 2σ(*F*^2^)] = 0.032	Hydrogen site location: inferred from neighbouring sites
*wR*(*F*^2^) = 0.077	H-atom parameters constrained
*S* = 1.04	*w* = 1/[σ^2^(*F*_o_^2^) + (0.0409*P*)^2^] where *P* = (*F*_o_^2^ + 2*F*_c_^2^)/3
4802 reflections	(Δ/σ)_max_ < 0.001
317 parameters	Δρ_max_ = 0.92 e Å^−^^3^
0 restraints	Δρ_min_ = −0.86 e Å^−^^3^

### Special details

**Table:**

Experimental. Spectroscopic data for the title compound: ^1^H NMR (CDCl_3_): *δ* 5.26 (s, 4H, C_5_*H*_2_), 1.33 (s, 18H, C(C*H*_3_)_3_), 0.44 (s, 6H, Si(C*H*_3_)), 0.23 (s, 6H, Si(C*H*_3_)). IR (*ν*_CO_): 2016(*s*), 1964(*s*), 1953(*s*), 1918(*s*) cm^-1^.
Geometry. Bond distances, angles etc. have been calculated using the rounded fractional coordinates. All su's are estimated from the variances of the (full) variance-covariance matrix. The cell esds are taken into account in the estimation of distances, angles and torsion angles
Refinement. Refinement of *F*^2^ against ALL reflections. The weighted *R*-factor *wR* and goodness of fit *S* are based on *F*^2^, conventional *R*-factors *R* are based on *F*, with *F* set to zero for negative *F*^2^. The threshold expression of *F*^2^ > σ(*F*^2^) is used only for calculating *R*-factors(gt) *etc*. and is not relevant to the choice of reflections for refinement. *R*-factors based on *F*^2^ are statistically about twice as large as those based on *F*, and *R*- factors based on ALL data will be even larger.

### Fractional atomic coordinates and isotropic or equivalent isotropic displacement parameters (Å^2^)

**Table:**

	*x*	*y*	*z*	*U*_iso_*/*U*_eq_	
Ru1	0.18780 (3)	0.25054 (2)	0.24573 (2)	0.0120 (1)	
Ru2	−0.01860 (3)	0.14546 (3)	0.30871 (2)	0.0119 (1)	
Si1	0.05491 (10)	0.22259 (9)	0.06637 (7)	0.0147 (2)	
Si2	−0.20390 (10)	0.49039 (9)	0.27597 (7)	0.0155 (3)	
O1	0.2229 (3)	0.2477 (3)	0.44361 (19)	0.0323 (9)	
O2	0.4503 (3)	−0.0461 (2)	0.16495 (19)	0.0268 (8)	
O3	−0.0771 (3)	0.2370 (3)	0.51910 (18)	0.0369 (9)	
O4	0.2411 (3)	−0.1387 (2)	0.3097 (2)	0.0320 (9)	
C1	0.1049 (4)	0.3370 (3)	0.1239 (2)	0.0144 (9)	
C2	0.0009 (4)	0.4414 (3)	0.2140 (2)	0.0135 (9)	
C3	0.0875 (3)	0.4865 (3)	0.2459 (2)	0.0140 (9)	
C4	0.2440 (4)	0.4151 (3)	0.1790 (2)	0.0147 (9)	
C5	0.2514 (4)	0.3265 (3)	0.1043 (2)	0.0148 (9)	
C6	0.3673 (4)	0.4458 (3)	0.1819 (2)	0.0144 (9)	
C7	0.3545 (4)	0.4787 (4)	0.2878 (2)	0.0205 (11)	
C8	0.3461 (4)	0.5772 (3)	0.1329 (3)	0.0222 (11)	
C9	0.5264 (4)	0.3196 (3)	0.1258 (3)	0.0204 (10)	
C10	−0.0830 (3)	0.2036 (3)	0.1740 (2)	0.0138 (9)	
C11	−0.0919 (3)	0.0804 (3)	0.1964 (2)	0.0124 (9)	
C12	−0.2045 (3)	0.1098 (3)	0.2926 (2)	0.0152 (9)	
C13	−0.2670 (3)	0.2564 (3)	0.3280 (2)	0.0141 (9)	
C14	−0.1938 (3)	0.3164 (3)	0.2584 (2)	0.0141 (9)	
C15	−0.2601 (4)	0.0125 (3)	0.3415 (2)	0.0153 (9)	
C16	−0.3307 (4)	0.0588 (4)	0.4535 (2)	0.0262 (11)	
C17	−0.3792 (4)	0.0198 (4)	0.3014 (3)	0.0267 (11)	
C18	−0.1273 (4)	−0.1413 (3)	0.3170 (3)	0.0210 (10)	
C19	−0.0376 (4)	0.3094 (4)	−0.0237 (3)	0.0242 (11)	
C20	0.2232 (4)	0.0455 (3)	0.0048 (2)	0.0220 (10)	
C21	−0.2787 (4)	0.5623 (3)	0.4091 (3)	0.0237 (11)	
C22	−0.3234 (4)	0.6240 (3)	0.2140 (3)	0.0261 (11)	
C23	0.2077 (4)	0.2457 (3)	0.3686 (3)	0.0219 (11)	
C24	0.3476 (4)	0.0650 (3)	0.1998 (2)	0.0165 (10)	
C25	0.1445 (4)	−0.0298 (3)	0.3083 (3)	0.0189 (10)	
C26	−0.0499 (4)	0.2012 (4)	0.4378 (3)	0.0226 (11)	
H3	0.04540	0.55950	0.30500	0.0170*	
H5	0.34450	0.26760	0.04500	0.0180*	
H7A	0.25240	0.55820	0.32450	0.0310*	
H7B	0.43190	0.50370	0.28730	0.0310*	
H7C	0.37120	0.39500	0.31920	0.0310*	
H8A	0.35170	0.55850	0.06550	0.0330*	
H8B	0.42700	0.59750	0.13150	0.0330*	
H8C	0.24610	0.65890	0.17040	0.0330*	
H9A	0.53660	0.23440	0.15360	0.0310*	
H9B	0.60470	0.33920	0.13170	0.0310*	
H9C	0.53960	0.30440	0.05630	0.0310*	
H11	−0.03090	−0.01300	0.15120	0.0150*	
H13	−0.35290	0.31030	0.39170	0.0170*	
H16A	−0.25520	0.05990	0.47790	0.0390*	
H16B	−0.36150	−0.00790	0.48400	0.0390*	
H16C	−0.42060	0.15410	0.47030	0.0390*	
H17A	−0.46140	0.11850	0.31340	0.0400*	
H17B	−0.42160	−0.03800	0.33450	0.0400*	
H17C	−0.33110	−0.01620	0.23060	0.0400*	
H18A	−0.08330	−0.17310	0.24570	0.0310*	
H18B	−0.16540	−0.20260	0.34930	0.0310*	
H18C	−0.04840	−0.14630	0.34060	0.0310*	
H19A	0.03670	0.31820	−0.07970	0.0370*	
H19B	−0.12490	0.40380	0.00820	0.0370*	
H19C	−0.07320	0.25180	−0.04680	0.0370*	
H20A	0.30570	0.05720	−0.04050	0.0330*	
H20B	0.19430	−0.00550	−0.03200	0.0330*	
H20C	0.25830	−0.00850	0.05440	0.0330*	
H21A	−0.37670	0.56560	0.44120	0.0360*	
H21B	−0.29370	0.65810	0.41720	0.0360*	
H21C	−0.20530	0.50100	0.43920	0.0360*	
H22A	−0.28710	0.58440	0.14440	0.0390*	
H22B	−0.31540	0.70950	0.21930	0.0390*	
H22C	−0.43040	0.64830	0.24560	0.0390*	

### Atomic displacement parameters (Å^2^)

**Table:**

	*U*^11^	*U*^22^	*U*^33^	*U*^12^	*U*^13^	*U*^23^
Ru1	0.0114 (1)	0.0114 (1)	0.0144 (2)	−0.0058 (1)	−0.0059 (1)	0.0043 (1)
Ru2	0.0112 (1)	0.0122 (1)	0.0127 (2)	−0.0060 (1)	−0.0045 (1)	0.0041 (1)
Si1	0.0167 (4)	0.0169 (4)	0.0132 (4)	−0.0095 (4)	−0.0069 (4)	0.0049 (4)
Si2	0.0113 (4)	0.0105 (4)	0.0237 (5)	−0.0045 (3)	−0.0067 (4)	0.0036 (4)
O1	0.0476 (17)	0.0405 (16)	0.0266 (15)	−0.0297 (14)	−0.0226 (14)	0.0152 (13)
O2	0.0179 (12)	0.0184 (13)	0.0359 (15)	−0.0051 (11)	−0.0060 (11)	0.0046 (11)
O3	0.0342 (15)	0.0594 (19)	0.0155 (14)	−0.0219 (14)	−0.0090 (12)	0.0016 (13)
O4	0.0219 (13)	0.0200 (13)	0.0533 (18)	−0.0085 (12)	−0.0159 (13)	0.0177 (12)
C1	0.0159 (16)	0.0147 (16)	0.0168 (17)	−0.0103 (13)	−0.0066 (14)	0.0086 (13)
C2	0.0137 (15)	0.0096 (15)	0.0185 (17)	−0.0062 (13)	−0.0068 (14)	0.0058 (13)
C3	0.0158 (16)	0.0088 (15)	0.0181 (17)	−0.0057 (13)	−0.0075 (14)	0.0032 (13)
C4	0.0181 (16)	0.0127 (15)	0.0146 (16)	−0.0086 (13)	−0.0060 (14)	0.0071 (13)
C5	0.0187 (16)	0.0167 (16)	0.0133 (16)	−0.0118 (14)	−0.0060 (14)	0.0055 (13)
C6	0.0159 (16)	0.0169 (16)	0.0152 (16)	−0.0109 (14)	−0.0068 (14)	0.0031 (13)
C7	0.0236 (18)	0.0253 (18)	0.0234 (19)	−0.0166 (15)	−0.0143 (16)	0.0055 (15)
C8	0.0194 (17)	0.0210 (18)	0.032 (2)	−0.0123 (15)	−0.0127 (16)	0.0119 (16)
C9	0.0161 (17)	0.0212 (17)	0.0256 (19)	−0.0099 (14)	−0.0087 (15)	0.0048 (15)
C10	0.0140 (16)	0.0133 (15)	0.0169 (17)	−0.0061 (13)	−0.0102 (14)	0.0053 (13)
C11	0.0127 (15)	0.0132 (15)	0.0158 (16)	−0.0080 (13)	−0.0082 (13)	0.0025 (13)
C12	0.0127 (15)	0.0160 (16)	0.0176 (17)	−0.0066 (13)	−0.0070 (14)	0.0031 (13)
C13	0.0082 (14)	0.0137 (16)	0.0215 (17)	−0.0046 (13)	−0.0080 (13)	0.0031 (13)
C14	0.0099 (15)	0.0139 (16)	0.0175 (17)	−0.0040 (13)	−0.0068 (13)	0.0048 (13)
C15	0.0157 (16)	0.0126 (15)	0.0196 (17)	−0.0087 (13)	−0.0062 (14)	0.0065 (13)
C16	0.032 (2)	0.0204 (18)	0.0217 (19)	−0.0150 (16)	−0.0009 (16)	0.0039 (15)
C17	0.0237 (19)	0.029 (2)	0.039 (2)	−0.0179 (16)	−0.0178 (17)	0.0178 (17)
C18	0.0185 (17)	0.0165 (17)	0.0252 (19)	−0.0086 (14)	−0.0047 (15)	0.0088 (15)
C19	0.031 (2)	0.030 (2)	0.0216 (18)	−0.0191 (17)	−0.0154 (16)	0.0109 (16)
C20	0.0219 (18)	0.0234 (18)	0.0202 (18)	−0.0117 (15)	−0.0061 (15)	−0.0003 (15)
C21	0.0208 (18)	0.0196 (18)	0.030 (2)	−0.0114 (15)	−0.0060 (16)	−0.0016 (15)
C22	0.0214 (18)	0.0153 (17)	0.045 (2)	−0.0094 (15)	−0.0155 (17)	0.0089 (16)
C23	0.0251 (19)	0.0179 (17)	0.028 (2)	−0.0140 (15)	−0.0106 (17)	0.0082 (15)
C24	0.0160 (17)	0.0173 (17)	0.0200 (17)	−0.0100 (15)	−0.0084 (14)	0.0096 (14)
C25	0.0195 (17)	0.0229 (18)	0.0228 (18)	−0.0156 (16)	−0.0097 (15)	0.0104 (15)
C26	0.0160 (17)	0.0288 (19)	0.025 (2)	−0.0124 (15)	−0.0079 (16)	0.0075 (16)

### Geometric parameters (Å, º)

**Table:**

Ru1—Ru2	2.8401 (11)	C12—C15	1.515 (5)
Ru1—C1	2.230 (3)	C13—C14	1.426 (4)
Ru1—C2	2.260 (4)	C15—C16	1.534 (4)
Ru1—C3	2.264 (3)	C15—C17	1.523 (7)
Ru1—C4	2.265 (4)	C15—C18	1.538 (5)
Ru1—C5	2.236 (3)	C3—H3	1.0000
Ru1—C23	1.863 (4)	C5—H5	1.0000
Ru1—C24	1.857 (3)	C7—H7A	0.9800
Ru2—C10	2.263 (3)	C7—H7B	0.9800
Ru2—C11	2.264 (3)	C7—H7C	0.9800
Ru2—C12	2.267 (4)	C8—H8A	0.9800
Ru2—C13	2.239 (4)	C8—H8B	0.9800
Ru2—C14	2.237 (3)	C8—H8C	0.9800
Ru2—C25	1.868 (3)	C9—H9A	0.9800
Ru2—C26	1.854 (4)	C9—H9B	0.9800
Si1—C1	1.861 (4)	C9—H9C	0.9800
Si1—C10	1.865 (3)	C11—H11	1.0000
Si1—C19	1.867 (4)	C13—H13	1.0000
Si1—C20	1.858 (3)	C16—H16A	0.9800
Si2—C2	1.871 (4)	C16—H16B	0.9800
Si2—C14	1.861 (3)	C16—H16C	0.9800
Si2—C21	1.859 (4)	C17—H17A	0.9800
Si2—C22	1.867 (4)	C17—H17B	0.9800
O1—C23	1.154 (5)	C17—H17C	0.9800
O2—C24	1.147 (4)	C18—H18A	0.9800
O3—C26	1.148 (5)	C18—H18B	0.9800
O4—C25	1.146 (4)	C18—H18C	0.9800
C1—C2	1.464 (4)	C19—H19A	0.9800
C1—C5	1.439 (6)	C19—H19B	0.9800
C2—C3	1.417 (6)	C19—H19C	0.9800
C3—C4	1.441 (5)	C20—H20A	0.9800
C4—C5	1.417 (5)	C20—H20B	0.9800
C4—C6	1.511 (6)	C20—H20C	0.9800
C6—C7	1.533 (4)	C21—H21A	0.9800
C6—C8	1.543 (4)	C21—H21B	0.9800
C6—C9	1.532 (5)	C21—H21C	0.9800
C10—C11	1.417 (4)	C22—H22A	0.9800
C10—C14	1.462 (4)	C22—H22B	0.9800
C11—C12	1.441 (4)	C22—H22C	0.9800
C12—C13	1.429 (4)		
			
Ru2—Ru1—C1	92.46 (11)	Si1—C10—C14	123.8 (2)
Ru2—Ru1—C2	87.88 (11)	C11—C10—C14	107.0 (3)
Ru2—Ru1—C3	117.71 (9)	Ru2—C11—C10	71.73 (18)
Ru2—Ru1—C4	150.11 (11)	Ru2—C11—C12	71.53 (18)
Ru2—Ru1—C5	127.88 (12)	C10—C11—C12	110.4 (2)
Ru2—Ru1—C23	90.24 (14)	Ru2—C12—C11	71.4 (2)
Ru2—Ru1—C24	88.90 (13)	Ru2—C12—C13	70.5 (2)
C1—Ru1—C2	38.06 (10)	Ru2—C12—C15	128.1 (2)
C1—Ru1—C3	62.19 (11)	C11—C12—C13	105.4 (3)
C1—Ru1—C4	62.96 (14)	C11—C12—C15	127.8 (3)
C1—Ru1—C5	37.60 (16)	C13—C12—C15	126.5 (3)
C1—Ru1—C23	159.41 (12)	Ru2—C13—C12	72.6 (2)
C1—Ru1—C24	108.96 (12)	Ru2—C13—C14	71.4 (2)
C2—Ru1—C3	36.51 (14)	C12—C13—C14	110.6 (3)
C2—Ru1—C4	62.34 (15)	Ru2—C14—Si2	113.04 (17)
C2—Ru1—C5	62.11 (13)	Ru2—C14—C10	72.03 (17)
C2—Ru1—C23	121.78 (13)	Ru2—C14—C13	71.48 (18)
C2—Ru1—C24	146.58 (13)	Si2—C14—C10	124.1 (2)
C3—Ru1—C4	37.11 (12)	Si2—C14—C13	128.4 (2)
C3—Ru1—C5	60.99 (10)	C10—C14—C13	106.6 (3)
C3—Ru1—C23	98.65 (12)	C12—C15—C16	110.7 (3)
C3—Ru1—C24	151.24 (14)	C12—C15—C17	107.6 (3)
C4—Ru1—C5	36.70 (12)	C12—C15—C18	110.7 (3)
C4—Ru1—C23	107.05 (15)	C16—C15—C17	110.2 (3)
C4—Ru1—C24	114.14 (15)	C16—C15—C18	108.8 (3)
C5—Ru1—C23	141.38 (17)	C17—C15—C18	108.9 (3)
C5—Ru1—C24	94.77 (12)	Ru1—C23—O1	176.4 (4)
C23—Ru1—C24	91.48 (14)	Ru1—C24—O2	174.7 (3)
Ru1—Ru2—C10	88.92 (9)	Ru2—C25—O4	177.7 (4)
Ru1—Ru2—C11	118.88 (8)	Ru2—C26—O3	176.4 (4)
Ru1—Ru2—C12	151.16 (7)	Ru1—C3—H3	125.00
Ru1—Ru2—C13	127.78 (8)	C2—C3—H3	125.00
Ru1—Ru2—C14	93.14 (9)	C4—C3—H3	125.00
Ru1—Ru2—C25	89.05 (14)	Ru1—C5—H5	125.00
Ru1—Ru2—C26	88.82 (15)	C1—C5—H5	125.00
C10—Ru2—C11	36.47 (11)	C4—C5—H5	125.00
C10—Ru2—C12	62.40 (11)	C6—C7—H7A	109.00
C10—Ru2—C13	61.87 (11)	C6—C7—H7B	109.00
C10—Ru2—C14	37.90 (10)	C6—C7—H7C	109.00
C10—Ru2—C25	122.62 (15)	H7A—C7—H7B	110.00
C10—Ru2—C26	148.19 (15)	H7A—C7—H7C	110.00
C11—Ru2—C12	37.10 (11)	H7B—C7—H7C	109.00
C11—Ru2—C13	60.93 (11)	C6—C8—H8A	109.00
C11—Ru2—C14	61.88 (11)	C6—C8—H8B	110.00
C11—Ru2—C25	100.00 (15)	C6—C8—H8C	110.00
C11—Ru2—C26	150.71 (17)	H8A—C8—H8B	109.00
C12—Ru2—C13	36.97 (11)	H8A—C8—H8C	109.00
C12—Ru2—C14	62.81 (12)	H8B—C8—H8C	110.00
C12—Ru2—C25	108.32 (16)	C6—C9—H9A	109.00
C12—Ru2—C26	113.61 (17)	C6—C9—H9B	109.00
C13—Ru2—C14	37.15 (11)	C6—C9—H9C	109.00
C13—Ru2—C25	142.88 (16)	H9A—C9—H9B	109.00
C13—Ru2—C26	95.42 (16)	H9A—C9—H9C	109.00
C14—Ru2—C25	160.22 (15)	H9B—C9—H9C	110.00
C14—Ru2—C26	110.62 (15)	Ru2—C11—H11	125.00
C25—Ru2—C26	89.06 (18)	C10—C11—H11	125.00
C1—Si1—C10	102.63 (14)	C12—C11—H11	125.00
C1—Si1—C19	111.50 (17)	Ru2—C13—H13	125.00
C1—Si1—C20	112.56 (19)	C12—C13—H13	125.00
C10—Si1—C19	110.73 (19)	C14—C13—H13	125.00
C10—Si1—C20	109.94 (14)	C15—C16—H16A	109.00
C19—Si1—C20	109.34 (16)	C15—C16—H16B	109.00
C2—Si2—C14	101.74 (15)	C15—C16—H16C	110.00
C2—Si2—C21	112.61 (19)	H16A—C16—H16B	109.00
C2—Si2—C22	109.47 (17)	H16A—C16—H16C	109.00
C14—Si2—C21	110.45 (14)	H16B—C16—H16C	109.00
C14—Si2—C22	113.01 (17)	C15—C17—H17A	109.00
C21—Si2—C22	109.43 (17)	C15—C17—H17B	109.00
Ru1—C1—Si1	112.78 (14)	C15—C17—H17C	109.00
Ru1—C1—C2	72.10 (18)	H17A—C17—H17B	110.00
Ru1—C1—C5	71.42 (19)	H17A—C17—H17C	109.00
Si1—C1—C2	123.5 (3)	H17B—C17—H17C	110.00
Si1—C1—C5	129.5 (2)	C15—C18—H18A	110.00
C2—C1—C5	106.0 (3)	C15—C18—H18B	109.00
Ru1—C2—Si2	120.17 (15)	C15—C18—H18C	109.00
Ru1—C2—C1	69.84 (18)	H18A—C18—H18B	110.00
Ru1—C2—C3	71.9 (2)	H18A—C18—H18C	109.00
Si2—C2—C1	124.1 (3)	H18B—C18—H18C	109.00
Si2—C2—C3	128.5 (2)	Si1—C19—H19A	109.00
C1—C2—C3	107.3 (3)	Si1—C19—H19B	109.00
Ru1—C3—C2	71.62 (17)	Si1—C19—H19C	109.00
Ru1—C3—C4	71.51 (17)	H19A—C19—H19B	109.00
C2—C3—C4	110.1 (3)	H19A—C19—H19C	110.00
Ru1—C4—C3	71.38 (19)	H19B—C19—H19C	109.00
Ru1—C4—C5	70.51 (19)	Si1—C20—H20A	109.00
Ru1—C4—C6	129.1 (2)	Si1—C20—H20B	109.00
C3—C4—C5	106.0 (4)	Si1—C20—H20C	109.00
C3—C4—C6	125.9 (3)	H20A—C20—H20B	109.00
C5—C4—C6	127.6 (3)	H20A—C20—H20C	109.00
Ru1—C5—C1	70.98 (17)	H20B—C20—H20C	110.00
Ru1—C5—C4	72.79 (17)	Si2—C21—H21A	109.00
C1—C5—C4	110.5 (3)	Si2—C21—H21B	110.00
C4—C6—C7	111.5 (3)	Si2—C21—H21C	109.00
C4—C6—C8	107.7 (3)	H21A—C21—H21B	109.00
C4—C6—C9	110.7 (3)	H21A—C21—H21C	109.00
C7—C6—C8	108.5 (3)	H21B—C21—H21C	109.00
C7—C6—C9	109.0 (3)	Si2—C22—H22A	109.00
C8—C6—C9	109.5 (3)	Si2—C22—H22B	110.00
Ru2—C10—Si1	118.11 (18)	Si2—C22—H22C	110.00
Ru2—C10—C11	71.80 (17)	H22A—C22—H22B	109.00
Ru2—C10—C14	70.08 (16)	H22A—C22—H22C	109.00
Si1—C10—C11	128.8 (2)	H22B—C22—H22C	109.00
			
C1—Ru1—Ru2—C10	7.43 (11)	C26—Ru2—C11—C10	120.7 (3)
C1—Ru1—Ru2—C11	29.18 (12)	C26—Ru2—C11—C12	0.9 (4)
C1—Ru1—Ru2—C12	1.64 (17)	Ru1—Ru2—C12—C11	42.2 (2)
C1—Ru1—Ru2—C13	−44.89 (12)	Ru1—Ru2—C12—C13	−72.5 (2)
C1—Ru1—Ru2—C14	−30.24 (10)	Ru1—Ru2—C12—C15	165.97 (16)
C1—Ru1—Ru2—C25	130.09 (15)	C10—Ru2—C12—C11	35.62 (16)
C1—Ru1—Ru2—C26	−140.84 (15)	C10—Ru2—C12—C13	−79.05 (17)
C2—Ru1—Ru2—C10	45.23 (10)	C10—Ru2—C12—C15	159.4 (3)
C2—Ru1—Ru2—C11	66.97 (11)	C11—Ru2—C12—C13	−114.7 (2)
C2—Ru1—Ru2—C12	39.44 (17)	C11—Ru2—C12—C15	123.8 (3)
C2—Ru1—Ru2—C13	−7.10 (12)	C13—Ru2—C12—C11	114.7 (2)
C2—Ru1—Ru2—C14	7.55 (10)	C13—Ru2—C12—C15	−121.5 (3)
C2—Ru1—Ru2—C25	167.88 (15)	C14—Ru2—C12—C11	78.52 (17)
C2—Ru1—Ru2—C26	−103.04 (15)	C14—Ru2—C12—C13	−36.14 (16)
C3—Ru1—Ru2—C10	67.10 (11)	C14—Ru2—C12—C15	−157.7 (3)
C3—Ru1—Ru2—C11	88.85 (12)	C25—Ru2—C12—C11	−82.3 (2)
C3—Ru1—Ru2—C12	61.31 (17)	C25—Ru2—C12—C13	163.07 (19)
C3—Ru1—Ru2—C13	14.78 (12)	C25—Ru2—C12—C15	41.6 (3)
C3—Ru1—Ru2—C14	29.43 (11)	C26—Ru2—C12—C11	−179.50 (19)
C3—Ru1—Ru2—C25	−170.24 (15)	C26—Ru2—C12—C13	65.8 (2)
C3—Ru1—Ru2—C26	−81.16 (15)	C26—Ru2—C12—C15	−55.7 (3)
C4—Ru1—Ru2—C10	40.45 (16)	Ru1—Ru2—C13—C12	144.40 (13)
C4—Ru1—Ru2—C11	62.19 (17)	Ru1—Ru2—C13—C14	24.7 (2)
C4—Ru1—Ru2—C12	34.7 (2)	C10—Ru2—C13—C12	80.59 (17)
C4—Ru1—Ru2—C13	−11.88 (17)	C10—Ru2—C13—C14	−39.09 (17)
C4—Ru1—Ru2—C14	2.77 (16)	C11—Ru2—C13—C12	38.84 (16)
C4—Ru1—Ru2—C25	163.10 (19)	C11—Ru2—C13—C14	−80.84 (18)
C4—Ru1—Ru2—C26	−107.82 (19)	C12—Ru2—C13—C14	−119.7 (2)
C5—Ru1—Ru2—C10	−6.32 (12)	C14—Ru2—C13—C12	119.7 (2)
C5—Ru1—Ru2—C11	15.43 (13)	C25—Ru2—C13—C12	−27.3 (3)
C5—Ru1—Ru2—C12	−12.11 (18)	C25—Ru2—C13—C14	−146.9 (3)
C5—Ru1—Ru2—C13	−58.64 (13)	C26—Ru2—C13—C12	−122.88 (19)
C5—Ru1—Ru2—C14	−43.99 (12)	C26—Ru2—C13—C14	117.4 (2)
C5—Ru1—Ru2—C25	116.34 (16)	Ru1—Ru2—C14—Si2	−36.01 (14)
C5—Ru1—Ru2—C26	−154.58 (16)	Ru1—Ru2—C14—C10	84.19 (18)
C23—Ru1—Ru2—C10	167.02 (13)	Ru1—Ru2—C14—C13	−160.67 (16)
C23—Ru1—Ru2—C11	−171.24 (13)	C10—Ru2—C14—Si2	−120.2 (3)
C23—Ru1—Ru2—C12	161.23 (18)	C10—Ru2—C14—C13	115.1 (3)
C23—Ru1—Ru2—C13	114.70 (14)	C11—Ru2—C14—Si2	−157.3 (2)
C23—Ru1—Ru2—C14	129.34 (12)	C11—Ru2—C14—C10	−37.08 (19)
C23—Ru1—Ru2—C25	−70.33 (16)	C11—Ru2—C14—C13	78.06 (19)
C23—Ru1—Ru2—C26	18.75 (16)	C12—Ru2—C14—Si2	160.63 (18)
C24—Ru1—Ru2—C10	−101.50 (12)	C12—Ru2—C14—C10	−79.2 (2)
C24—Ru1—Ru2—C11	−79.76 (13)	C12—Ru2—C14—C13	35.97 (17)
C24—Ru1—Ru2—C12	−107.29 (18)	C13—Ru2—C14—Si2	124.7 (2)
C24—Ru1—Ru2—C13	−153.83 (13)	C13—Ru2—C14—C10	−115.1 (3)
C24—Ru1—Ru2—C14	−139.18 (12)	C26—Ru2—C14—Si2	53.9 (2)
C24—Ru1—Ru2—C25	21.15 (16)	C26—Ru2—C14—C10	174.1 (2)
C24—Ru1—Ru2—C26	110.23 (16)	C26—Ru2—C14—C13	−70.8 (2)
Ru2—Ru1—C1—Si1	−36.22 (17)	C10—Si1—C1—Ru1	54.0 (2)
Ru2—Ru1—C1—C2	83.4 (2)	C10—Si1—C1—C2	−29.0 (3)
Ru2—Ru1—C1—C5	−162.10 (15)	C10—Si1—C1—C5	138.2 (3)
C2—Ru1—C1—Si1	−119.6 (3)	C19—Si1—C1—Ru1	172.57 (18)
C2—Ru1—C1—C5	114.5 (3)	C19—Si1—C1—C2	89.6 (3)
C3—Ru1—C1—Si1	−156.5 (2)	C19—Si1—C1—C5	−103.3 (3)
C3—Ru1—C1—C2	−36.8 (2)	C20—Si1—C1—Ru1	−64.1 (2)
C3—Ru1—C1—C5	77.66 (19)	C20—Si1—C1—C2	−147.1 (2)
C4—Ru1—C1—Si1	161.5 (2)	C20—Si1—C1—C5	20.0 (3)
C4—Ru1—C1—C2	−78.9 (2)	C1—Si1—C10—Ru2	−48.10 (19)
C4—Ru1—C1—C5	35.65 (17)	C1—Si1—C10—C11	−136.7 (3)
C5—Ru1—C1—Si1	125.9 (2)	C1—Si1—C10—C14	35.8 (3)
C5—Ru1—C1—C2	−114.5 (3)	C19—Si1—C10—Ru2	−167.21 (17)
C23—Ru1—C1—Si1	−133.5 (4)	C19—Si1—C10—C11	104.2 (3)
C23—Ru1—C1—C2	−13.9 (6)	C19—Si1—C10—C14	−83.4 (3)
C23—Ru1—C1—C5	100.7 (5)	C20—Si1—C10—Ru2	71.9 (2)
C24—Ru1—C1—Si1	53.5 (2)	C20—Si1—C10—C11	−16.7 (4)
C24—Ru1—C1—C2	173.1 (2)	C20—Si1—C10—C14	155.7 (3)
C24—Ru1—C1—C5	−72.4 (2)	C14—Si2—C2—Ru1	−47.3 (2)
Ru2—Ru1—C2—Si2	21.74 (17)	C14—Si2—C2—C1	37.6 (3)
Ru2—Ru1—C2—C1	−96.7 (2)	C14—Si2—C2—C3	−137.5 (3)
Ru2—Ru1—C2—C3	146.33 (16)	C21—Si2—C2—Ru1	70.9 (2)
C1—Ru1—C2—Si2	118.5 (3)	C21—Si2—C2—C1	155.9 (2)
C1—Ru1—C2—C3	−117.0 (3)	C21—Si2—C2—C3	−19.2 (3)
C3—Ru1—C2—Si2	−124.6 (2)	C22—Si2—C2—Ru1	−167.09 (18)
C3—Ru1—C2—C1	117.0 (3)	C22—Si2—C2—C1	−82.2 (3)
C4—Ru1—C2—Si2	−161.0 (2)	C22—Si2—C2—C3	102.8 (3)
C4—Ru1—C2—C1	80.6 (2)	C2—Si2—C14—Ru2	52.36 (17)
C4—Ru1—C2—C3	−36.36 (17)	C2—Si2—C14—C10	−30.9 (3)
C5—Ru1—C2—Si2	157.4 (2)	C2—Si2—C14—C13	136.4 (3)
C5—Ru1—C2—C1	38.9 (2)	C21—Si2—C14—Ru2	−67.4 (2)
C5—Ru1—C2—C3	−78.1 (2)	C21—Si2—C14—C10	−150.7 (3)
C23—Ru1—C2—Si2	−67.2 (3)	C21—Si2—C14—C13	16.6 (4)
C23—Ru1—C2—C1	174.3 (2)	C22—Si2—C14—Ru2	169.62 (17)
C23—Ru1—C2—C3	57.4 (3)	C22—Si2—C14—C10	86.3 (3)
C24—Ru1—C2—Si2	106.5 (3)	C22—Si2—C14—C13	−106.3 (3)
C24—Ru1—C2—C1	−11.9 (4)	Ru1—C1—C2—Si2	−113.5 (2)
C24—Ru1—C2—C3	−128.9 (3)	Ru1—C1—C2—C3	62.5 (2)
Ru2—Ru1—C3—C2	−38.75 (18)	Si1—C1—C2—Ru1	105.9 (2)
Ru2—Ru1—C3—C4	−158.25 (17)	Si1—C1—C2—Si2	−7.6 (4)
C1—Ru1—C3—C2	38.41 (19)	Si1—C1—C2—C3	168.4 (2)
C1—Ru1—C3—C4	−81.1 (2)	C5—C1—C2—Ru1	−63.8 (2)
C2—Ru1—C3—C4	−119.5 (3)	C5—C1—C2—Si2	−177.3 (2)
C4—Ru1—C3—C2	119.5 (3)	C5—C1—C2—C3	−1.3 (3)
C5—Ru1—C3—C2	81.4 (2)	Ru1—C1—C5—C4	−62.4 (2)
C5—Ru1—C3—C4	−38.1 (2)	Si1—C1—C5—Ru1	−104.6 (2)
C23—Ru1—C3—C2	−133.6 (2)	Si1—C1—C5—C4	−167.0 (2)
C23—Ru1—C3—C4	106.9 (2)	C2—C1—C5—Ru1	64.29 (19)
C24—Ru1—C3—C2	117.0 (3)	C2—C1—C5—C4	1.9 (3)
C24—Ru1—C3—C4	−2.5 (4)	Ru1—C2—C3—C4	61.5 (2)
Ru2—Ru1—C4—C3	41.2 (3)	Si2—C2—C3—Ru1	114.5 (2)
Ru2—Ru1—C4—C5	−74.2 (3)	Si2—C2—C3—C4	176.0 (2)
Ru2—Ru1—C4—C6	162.69 (17)	C1—C2—C3—Ru1	−61.20 (19)
C1—Ru1—C4—C3	78.8 (2)	C1—C2—C3—C4	0.3 (3)
C1—Ru1—C4—C5	−36.5 (2)	Ru1—C3—C4—C5	62.4 (2)
C1—Ru1—C4—C6	−159.6 (3)	Ru1—C3—C4—C6	−125.3 (3)
C2—Ru1—C4—C3	35.78 (18)	C2—C3—C4—Ru1	−61.6 (2)
C2—Ru1—C4—C5	−79.6 (2)	C2—C3—C4—C5	0.9 (3)
C2—Ru1—C4—C6	157.3 (3)	C2—C3—C4—C6	173.2 (3)
C3—Ru1—C4—C5	−115.4 (3)	Ru1—C4—C5—C1	61.3 (2)
C3—Ru1—C4—C6	121.5 (3)	C3—C4—C5—Ru1	−63.0 (2)
C5—Ru1—C4—C3	115.4 (3)	C3—C4—C5—C1	−1.7 (3)
C5—Ru1—C4—C6	−123.1 (4)	C6—C4—C5—Ru1	124.9 (3)
C23—Ru1—C4—C3	−81.7 (2)	C6—C4—C5—C1	−173.9 (3)
C23—Ru1—C4—C5	163.0 (2)	Ru1—C4—C6—C7	−56.1 (3)
C23—Ru1—C4—C6	39.8 (3)	Ru1—C4—C6—C8	−175.0 (2)
C24—Ru1—C4—C3	178.7 (2)	Ru1—C4—C6—C9	65.3 (3)
C24—Ru1—C4—C5	63.3 (3)	C3—C4—C6—C7	38.4 (4)
C24—Ru1—C4—C6	−59.8 (3)	C3—C4—C6—C8	−80.4 (3)
Ru2—Ru1—C5—C1	22.90 (19)	C3—C4—C6—C9	159.9 (3)
Ru2—Ru1—C5—C4	142.6 (2)	C5—C4—C6—C7	−150.9 (3)
C1—Ru1—C5—C4	119.7 (3)	C5—C4—C6—C8	90.3 (4)
C2—Ru1—C5—C1	−39.40 (18)	C5—C4—C6—C9	−29.4 (4)
C2—Ru1—C5—C4	80.3 (2)	Ru2—C10—C11—C12	61.4 (2)
C3—Ru1—C5—C1	−81.12 (19)	Si1—C10—C11—Ru2	111.8 (3)
C3—Ru1—C5—C4	38.6 (2)	Si1—C10—C11—C12	173.3 (3)
C4—Ru1—C5—C1	−119.7 (3)	C14—C10—C11—Ru2	−61.6 (2)
C23—Ru1—C5—C1	−146.4 (2)	C14—C10—C11—C12	−0.2 (4)
C23—Ru1—C5—C4	−26.7 (3)	Ru2—C10—C14—Si2	106.1 (3)
C24—Ru1—C5—C1	115.2 (2)	Ru2—C10—C14—C13	−63.6 (2)
C24—Ru1—C5—C4	−125.1 (3)	Si1—C10—C14—Ru2	−111.1 (3)
Ru1—Ru2—C10—Si1	22.01 (14)	Si1—C10—C14—Si2	−5.0 (4)
Ru1—Ru2—C10—C11	146.93 (18)	Si1—C10—C14—C13	−174.7 (2)
Ru1—Ru2—C10—C14	−96.51 (18)	C11—C10—C14—Ru2	62.7 (2)
C11—Ru2—C10—Si1	−124.9 (3)	C11—C10—C14—Si2	168.8 (2)
C11—Ru2—C10—C14	116.6 (3)	C11—C10—C14—C13	−0.9 (4)
C12—Ru2—C10—Si1	−161.1 (2)	Ru2—C11—C12—C13	62.7 (2)
C12—Ru2—C10—C11	−36.22 (18)	Ru2—C11—C12—C15	−124.2 (4)
C12—Ru2—C10—C14	80.3 (2)	C10—C11—C12—Ru2	−61.6 (2)
C13—Ru2—C10—Si1	156.8 (2)	C10—C11—C12—C13	1.1 (4)
C13—Ru2—C10—C11	−78.3 (2)	C10—C11—C12—C15	174.2 (3)
C13—Ru2—C10—C14	38.31 (19)	Ru2—C12—C13—C14	61.6 (3)
C14—Ru2—C10—Si1	118.5 (3)	C11—C12—C13—Ru2	−63.3 (2)
C14—Ru2—C10—C11	−116.6 (3)	C11—C12—C13—C14	−1.7 (4)
C25—Ru2—C10—Si1	−66.2 (2)	C15—C12—C13—Ru2	123.5 (3)
C25—Ru2—C10—C11	58.7 (3)	C15—C12—C13—C14	−174.9 (3)
C25—Ru2—C10—C14	175.3 (2)	Ru2—C12—C15—C16	63.2 (4)
C26—Ru2—C10—Si1	108.0 (3)	Ru2—C12—C15—C17	−176.4 (2)
C26—Ru2—C10—C11	−127.1 (3)	Ru2—C12—C15—C18	−57.5 (3)
C26—Ru2—C10—C14	−10.5 (4)	C11—C12—C15—C16	158.5 (4)
Ru1—Ru2—C11—C10	−38.5 (2)	C11—C12—C15—C17	−81.0 (4)
Ru1—Ru2—C11—C12	−158.30 (14)	C11—C12—C15—C18	37.8 (5)
C10—Ru2—C11—C12	−119.8 (3)	C13—C12—C15—C16	−29.7 (5)
C12—Ru2—C11—C10	119.8 (3)	C13—C12—C15—C17	90.7 (4)
C13—Ru2—C11—C10	81.06 (19)	C13—C12—C15—C18	−150.4 (3)
C13—Ru2—C11—C12	−38.71 (17)	Ru2—C13—C14—Si2	−105.2 (3)
C14—Ru2—C11—C10	38.53 (18)	Ru2—C13—C14—C10	64.0 (2)
C14—Ru2—C11—C12	−81.23 (18)	C12—C13—C14—Ru2	−62.3 (2)
C25—Ru2—C11—C10	−133.0 (2)	C12—C13—C14—Si2	−167.5 (3)
C25—Ru2—C11—C12	107.2 (2)	C12—C13—C14—C10	1.6 (4)

### Hydrogen-bond geometry (Å, º)

**Table:**

*D*—H···*A*	*D*—H	H···*A*	*D*···*A*	*D*—H···*A*
C20—H20*A*···O2^i^	0.98	2.60	3.571 (5)	171

Symmetry code: (i) −*x*+1, −*y*, −*z*.

## References

[bb1] Bitterwolf, T. E., Leonard, M. B., Horine, P. A., Shade, J. E., Rheingold, A. L., Staley, D. J. & Yap, G. P. A. (1996). *J. Organomet. Chem.* **512**, 11–20.

[bb2] Boese, R., Cammack, J. K., Matzger, A. J., Pflug, K., Tolman, W. B., Vollhardt, K. P. C. & Weidman, T. W. (1997). *J. Am. Chem. Soc.* **119**, 6757–6773.

[bb3] Bruker (2005). *APEX2*, *SAINT* and *SADABS* Bruker AXS Inc., Madison, Wisconsin, USA.

[bb4] Burger, P. (2001). *Angew. Chem. Int. Ed.* **40**, 1917–1919.

[bb5] Mills, O. S. & Nice, J. P. (1967). *J. Organomet. Chem.* **9**, 339–344.

[bb6] Ovchinnikov, M. V., Klein, D. P., Guzei, I. A., Choi, M. G. & Angelici, R. J. (2002). *Organometallics*, **21**, 617–627.

[bb7] Sheldrick, G. M. (2008). *Acta Cryst.* A**64**, 112–122. 10.1107/S0108767307043930 18156677

[bb8] Zhou, X., Zhang, Y., Xu, S., Tian, G. & Wang, B. (1997). *Inorg. Chim. Acta*, **262**, 109–112.

[bb9] Zhu, B., Xu, S., Zhou, X. & Wang, B. (2012). *J. Organomet. Chem.* **708–709**, 88–97.

